# The Impact of Foreign Direct Investment on Environment Degradation: Evidence from Emerging Markets in Asia

**DOI:** 10.3390/ijerph16091636

**Published:** 2019-05-10

**Authors:** Anh Hoang To, Dao Thi-Thieu Ha, Ha Minh Nguyen, Duc Hong Vo

**Affiliations:** 1 Vietnam—the Netherlands Economics Program, Ho Chi Minh City 700000, Vietnam; anh.th@vnp.edu.vn; 2 International Economics Faculty, Banking University, Ho Chi Minh City 700000, Vietnam; daohtt@buh.edu.vn; 3 Business and Economics Research Group; HCMC Open University, Ho Chi Minh City 722000, Vietnam; ha.nm@ou.edu.vn

**Keywords:** FDI, environment degradation, pollution heaven hypothesis FMOLS, DOLS, causality, Vietnam

## Abstract

This study is conducted to examine the concerns of the foreign direct investment (FDI) causing environment degradation and also to test the validity of the traditional Environmental Kuznets Curve (EKC) in the context of emerging markets in the Asian region. Data of these countries from 1980–2016 are utilised. This study employs panel cointegration Fully Modified Ordinary Least Squares (FMOLS), which treats the endogeneity problem, and its estimators are adjusted for serial correlation. Moreover, this study also uses panel Dynamic Ordinary Least Squares (DOLS), which includes contemporaneous value, leads and, lags of the first difference of the regressors to correct endogeneity problems and serial correlations. Findings from this study indicate that the pollution heaven hypothesis and the EKC curve are generally valid in the region. In addition, FDI has a strong impact on the environment.

## 1. Introduction

For recent decades, emerging markets in Asia have achieved significant economic growth. Their economic growth depends heavily on capital. The nations have attracted foreign direct investment (FDI), which has a positive impact on economic growth in host countries. Thus, FDI has been becoming a gradually more important source of capital that transfers management skills and technologies and generates job opportunities and makes incremental contributions to export activities, thus improving the standard of living for millions of people in the region. However, FDI also leads to environmental degradation for the host countries. Host countries considered the trade-off between environmental degradation and growth so that they are able to attract FDI to their countries. In practice, many incidents have occurred in relation to significant environmental damages for the host countries, such as the case of Formosa Chemicals Corporation in Vietnam in 2016—a significant degradation led to at least 115 tons of dead fish; 450 hectares of coral reefs were significantly destroyed and more than 350 hectares of shrimp farming were killed, affecting the living conditions and income of more than 226,000 local vulnerable people in Vietnam [1]. This issue raises a fundamental question for many governments to find out whether FDI always provides positive effects on the host countries or whether it leads to environmental degradation.

This study uses 25 emerging markets in the Asian region from the 1980–2016 period. Previous studies usually employed time series data for a specific country and the maximum of 5 countries is found in their analyses. In order to achieve robust evidence, this study provides evidence on the link among four fundamental determinants; they are FDI, CO_2_ emissions, economic growth, and oil consumption using panel data analysis method.

This study is different from the previous studies on the following grounds. First, previous studies have not put great effort in to analyzing the complexity of the relationship between FDI and CO_2_ emissions. This relationship is of importance because developing countries attract FDI by adopting relaxed environmental regulations. However, if the findings prove that FDI promotes clean technology with low CO_2_ emissions, it will be a great potential for those countries to attract more FDI. Second, previous papers have not taken into account the important source of CO_2_ emissions from the oil sector, whose usage has been increasing rapidly in developing countries recently. Third, this study uses a sample of 25 countries, one of the largest samples of its kind of analysis, to provide robust evidence.

## 2. Literature Review

### 2.1. Theoretical Background

The Kuznets curve hypothesis, which was first discussed by the economist Simon Kuznets in the 1950s, shows that as an economy grows, initial inequality increases and then decreases [2]. The Kuznets curve indicates that the economic center of the nation will shift to urban regions when the nation experiences industrialization, especially agricultural mechanization [2]. Therefore, farmers tend to flock into large cities in order to find better-paying jobs. This means that there is a considerable inequality gap between rural and urban areas. Because rural populations plunge while urban populations soar, firm owners earn more profit while laborers in these industries receive an income rise at a slower rate and farmer incomes reduce. However, the inequality then hopefully drops when economic growth reaches the highest point of average income and the well-being of the state, reaching from industrialization to democratization, allows beneficial growth, leading to an increase in GDP per capita. Kuznets shows that inequality would have a trend like an inverted U-shape as it first increases and then decreases along with the increase of GDP per capita. While Kuznets curve diagrams illustrate an inverted U-shape curve, several variables along the axes are usually mixed and matched, such as the Gini coefficient or inequality on the *Y*-axis and economic growth, time, or GPD per capita on the *X*-axis.

**Figure 1:**
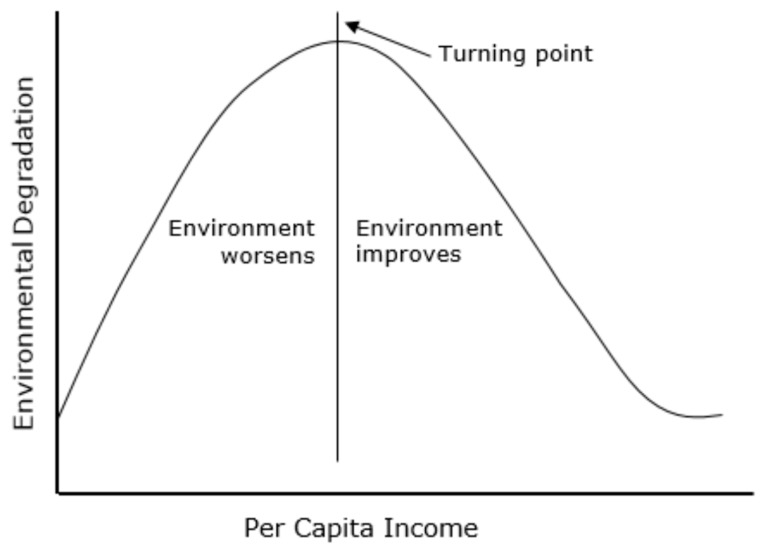
Environmental Kuznets curve.

After a few decades, Kruger and Grossman [3] applied this hypothesis in the environmental sector. It shows that economic development and environmental degradation is in an inverted U-shaped correlation which is known as the Environmental Kuznets Curve (EKC) theory. The EKC theory states that economic development and environmental degradation have a positive relationship. When the country is at an early stage of economic growth, improvement in living standards may be at the expense of the environment. However, when a high level of economic growth and development has been achieved, concerns about the environment emerge (Figure 1). As a result, the inverted U-shape of the EKC curve is supported by evidence from various empirical studies, including Shafik [4] and Omotor and Orubu [5]; it was considered a standard feature in the creation of environmental policy. Onafowora and Owoye’s research [6] showed that the long-run relationship between economic development and CO_2_ release follows an N-shape. Al-Mulali and Oxturk [7] indicated a U-shaped relationship between GDP and CO_2_ emission.

The second theory on the issue is generally known as the pollution heaven hypothesis, which states that polluting industries will be relocated to jurisdictions where environmental regulations are less stringent. There are two main arguments concerning the benefits that FDI brings to the economic development of host countries. First, FDI diminishes environmental degradation through technological innovation. Second, some have argued that FDIs take the environment issue more seriously, which increases CO_2_ emissions where pollution-intensive industries could be transferred from the rich to the poorer countries due to weak environmental law and regulations in the host countries. Similarly, in relation to economic growth and emission, this relationship is complicated and inconsistent. Findings from previous studies showed that FDI can have a positive or negative impact on the environment.

### 2.2. Empirical Literature

In general, there are three main schools of thought in relation to the causal relationship between FDI, economic growth, CO_2_ emissions, and energy consumption. The first stream is related to the empirical studies that mainly tested the validity of the Environmental Kuznets Curve (EKC) hypothesis. The second stream is the examination concentrating on the relationship between energy consumption and CO_2_ emissions. The final stream is the investigation of the hypothesis that there is a causal relationship between trade liberalization, which is represented by FDI flows, and CO_2_ emissions.

Utilizing panel cointegration with a pooled sample to test the EKC hypothesis, Lean and Smyth [8] concluded that, generally, for the whole sample of 5 Association of Southeast Asian Nations (ASEAN) countries, the EKC hypothesis is supported. However, at an individual country level, the relationship between economic growth and CO_2_ emissions varies. While the EKC hypothesis is supported in the Philippines, there is no support of EKC for Malaysia and Thailand. For Indonesia, income has a positive one-directional relationship with CO_2_ emissions. When panel Granger causality test is utilised, there is no causal relationship from income to CO_2_ emissions in both the short and the long run. However, there is a causal relationship between CO_2_ emissions and income in the long run.

However, EKC theory is not supported by other studies. Narayan and Narayan [9] investigated the EKC hypothesis by comparing the long and short run income elasticity of 43 developing countries and found that the EKC was not supported. Their findings for ASEAN-5 is that there is no evidence supporting the EKC for Indonesia, Malaysia, Philippines, and Thailand, while income in the Philippines has a positive impact on its CO_2_ emissions. This study shows that there is a long-run relationship between income and CO_2_ emissions.

Besides the test of the validity of traditional EKC theory (inverted U-shape), the new stream expanded into testing the existence of an N-shape relationship of income and CO_2_ emissions. This stream raised many empirical research questions for researchers such as Churchill et al. [10], Sarkodie and Strezov [11], and Zhou et al. [12]. Churchill et al. [10] tested N-shape for OECD countries in the period 1870–2014 by using mean group estimators (MG, PMG, AMG, and CCEMG), and found there are two turning points of GDP per capita or the relationship exhibits N-shape for some countries, such as Australia, Canada, and Japan, but some countries do not follow this shape, such as Spain and the UK. Moreover, Sarkodie and Strezov [11] also tested this N-shape for the top five greenhouse gas emitting developing countries—China, Iran, Indonesia, India, and South Africa—by using panel quantile regression with data from 1982 to 2016. The findings stated there is an N-shape of per capita income and CO_2_ emissions for selected countries and this still supports EKC theory [11].

Niu et al. [13] utilized panel data to analyze the causal relationships between GDP, energy consumption, and CO_2_ emissions for the Asia–Pacific countries, including developing countries such as China, India, Thailand, and Indonesia, and the results are mixed. While there is a long-run nexus between energy consumption, coal, oil, and CO_2_ emissions, there is no long-run nexus between natural gas and electricity on CO_2_ emissions. Like previous studies, they concluded that the energy consumption is the main cause of CO_2_ emissions. Moreover, the individual countries were examined by applying the individually-fixed varying coefficient model between CO_2_ emissions and the amount of energy used per capita [13]. In addition, Granger causality test showed that there is a causality between CO_2_ emissions and GDP, and energy consumption and CO_2_ emissions in the long run. Nevertheless, for the short-run analysis, it is significant for unidirectional causality between energy consumption and CO_2_ emissions.

Hossain M.S. [14] used panel data of 9 newly-industrialized nations, including Malaysia, the Philippines, and Thailand, to investigate the nexus between CO_2_ emissions, energy consumption, and economic growth as well as trade openness and urbanization. The paper found that income and energy consumption significantly impact on CO_2_ emissions in the long run for the Philippines and Thailand, whereas it is not significant for the case of Malaysia. Furthermore, the panel Granger causality test shows that there is no causal relationship between income, energy consumption, and CO_2_ emissions in the long run [14]. Nonetheless, in the short run, there was significant causality running from income to CO_2_ emissions.

More specifically, Ang [15] examined the relationship between GDP, CO_2_ emissions and energy consumption in the long run for the Malaysia case. The study showed that CO_2_ emissions and energy consumption have positive impacts on GDP in the long run. Like [14], the Granger causality test found evidence of unidirectional causality running from GDP to energy consumption in the long run. Similarly, the causality nexus between CO_2_ emissions and growth is weak and, therefore, it is inclusive.

There are a lot of empirical studies about the pollution heaven hypothesis (PHH) [16,17,18,19,20,21,22,23,24,25,26,27]; however, there are also conflicting findings between them. For instance, Sun et al. [16] used Auto Regressive Distributed Lag model (ARDL) to test this hypothesis for China; they confirmed the validity of PHH, but Zhang and Zhou [17], who also used it with Chinese data, found contrasting findings—a negative nexus between FDI and CO_2_ emissions. In addition, Zhu et al. [18] researched for 5 countries in Asia from 1981 to 2011 using panel quartile regression; they rejected the validity of PHH in these countries, while Behera and Dash [19] used data from 17 countries in Asia (including 5 countries from Zhu et al. [18]) from 1980 to 2012 and found contradictory results—a positive nexus between FDI and CO_2_ emissions; this supported the PHH. Furthermore, Tang and Tan [20], who used time series data in Vietnam from 1976 to 2009, found a negative nexus, while Phuong and Tuyen [21], also using data from Vietnam from 1986 to 2015, found no evidence on FDI and CO_2_ emissions. Moreover, a lot of previous empirical studies had similar conflicts in their findings. For the support side of PHH, Solarin et al. [22], who used the ARDL model with Ghanaian time series data, Zakarya et al. [23], who used panel data from 6 BRICS countries (Brazil, Russia, India, China, and South Africa) from 1990–2015, and Zhou et al. [12], who used data from 285 cities in China with GMM method for Random and Fixed Effect model, found a long-run positive relationship between FDI and CO_2_ emissions. For the rejected side, Kirkulak et al. [24], Acharya [25], and Jorgenson [26] found the negative nexus. In an opposite analysis, Atici [27] used panel data from 1970 to 2006 of ASEAN countries and Japan and found no evidence for this relationship.

In conclusion, previous studies show the complexity of the causal relationship between CO_2_ emissions, energy consumption, and economic growth [8,9,10,11,12,13,14,15,16,17,18,19,20,21,22,23,24,25,26,27]. The findings on these relationships are not robust. The evidence is inclusive and mixed among the studies. Moreover, the evidence on testing the validity of the EKC is also mixed in previous empirical studies. In addition, previous studies had not put great emphasis on analyzing the complexity of the relationship between FDI and CO_2_ emission. All these weaknesses are considered in this study.

## 3. Data and Methodology

### 3.1. Data and Variables

This study uses secondary data of 25 emerging markets and developing countries in the Asian region, Bangladesh, Bhutan, Brunei Darussalam, Cambodia, China, Fiji, India, Indonesia, Kiribati, Lao, Malaysia, Maldives, Mongolia, Macedonia, Myanmar, Nepal, Papua New Guinea, Philippines, Samoa, Solomon Islands, Sri Lanka, Thailand, Tonga, Vanuatu, and Vietnam, from the 1980–2016 period.

Table 1 shows the brief description of selected variables in the regression model. The details of expect signs and the reason for choosing these variables will be analyzed in the next section.

Table 2 shows a statistical overview of all the variables that are used for analysis. The distributions of all the variables are biased; Kurtosis statistic also points out that seven distributions are focused more than normal distributions with the help of long tails. All figures for these variables are presented in Appendix A.

### 3.2. Methodology and Specification

From the traditional EKC theory (Kuznets, 1950s), the relationship between income and environmental degradation is non-linear and has an inverted U-shape [2,3]. This implies that when income increases, environmental degradation increases until income reaches the threshold, and then degradation decrease. Therefore, the form of the model could be shown as below:

Y = F(GDP, GDP^2^, X)

where Y is an environment indicator, with common proxy by carbon emissions or greenhouse gas emissions. In this paper, we use carbon emissions proxies for Y since the up-to-date data for greenhouse gas are shorter than for carbon emissions. As a result, we have good results and analysis.

GDP and GDP^2^ are the two traditional variables in EKC theory, but as we mentioned above, the recent trend in testing EKC concerns whether this nexus is N-shaped [10,11,12]. Thus, we add the cubic GDP to the model. As a result, we can simultaneously test the validity of EKC and the new shape for our selected developing countries, which has not been done before.

X are other explanatory variables. In this paper, we use per capita oil consumption, foreign direct investment (FDI), and square FDI for testing the non-linear nexus, which is less of a concern in previous studies. The regression model is proposed as follows:

CO_2 it_ = π_0_ + π_1_Oil_it_+ π_2_G_it_ + π_3_G^2^_it_+ π_4_G^3^_it_+ π_5_FDI_it_ + π_6_FDI^2^_it_+ ε_it_ (1)

where carbon emissions (CO_2_) is used as the dependent variable.

Various independent variables are utilized, including per capita oil consumption, the per capita real GDP, net inflows of foreign direct investment, and the square and cube of per capita real GDP. All variables are transformed into logarithm form.

For the explanation of the first variable—oil consumption: Within selected countries, the current economy is primarily based on the industrial orient economy. Thus, with an increase in oil use, emissions increase. As a result, the expected sign of oil is positive.

The model includes the cube of GDP for testing whether the impacts of these variables are N-shaped, which has just been stated in recent papers. If an N-shaped relationship between GDP and CO_2_ emissions exists, then the two turning points of GDP are calculated using the following formula:

(2) $$GDP_{1,2}^{*}=e^{\frac{-\pi_{3}\pm \sqrt{\pi_{3}^{2}-3\pi_{2}\pi_{4}}}{3\;\pi_{4}}}$$

If there is an inverted U-shaped relationship between FDI and CO_2_ emissions, then the threshold of FDI is calculated using the following formula:

(3) $$\mathrm{FDI}*=e^{\frac{-\pi_{5}}{2\pi_{6}}}$$

From Equation (1) above, we conduct an empirical study on the direction of causality between variables, especially FDI and CO_2_ emissions. There are basically three steps to testing the causal relationship between economic growth and consumption in panel data. Firstly, the integration in time series of economic and energy variables is tested and arranged in order. After that, the long-run relationships among the variables in question are examined by using panel cointegration tests. Granger [31,32] stated that the series are integrated in order because they are stationary after the first variance; therefore, linear combinations can exist by a feature through which the series are stationary and without differences; these are called cointegrated series. The next step after the integration of order is shown is examining the existing long-run relationship between the group of integrated variables in question by using cointegration analysis. While cointegration is discovered, the rest, with a lack of information on any long-run relationships among variables, can be treated by using a Vector Error Correction model (VECM) to investigate whether there is a stationary linear combination of nonstationary variables, which would indicate a long-run equilibrium relationship among the variables. Finally, the long-run direction of causal linkages among the variables is evaluated by employing dynamic panel causality tests.

#### 3.2.1. Test of Cross Sectional Dependence

Pesaran [33,34] points out that panel data are likely to display considerable cross-sectional dependence in error terms, due to unobserved and common shock factors. The effect of cross-sectional dependence is estimated variously and relies on unobserved common factors such as the nature of cross-sectional dependence as well as the magnitude of the correlations across cross-sections. The common effect of cross-sectional dependence leads to standard errors, which causes biases in estimation, whereas fixed-effects (FE) and random-effects (RE), although not efficient, have consistent estimators. For treating this problem, the use of the approach, which is proposed by Driscoll and Kraay [35], may not work, and the FE and RE estimators would be biased. Another method is the use of the instrumental variables (IVs) approach. But finding the IVs in practice is not easy. From Equation (1) above, T denotes the panel time dimension, N denotes the cross-sectional dimension, and *u_it_* denotes error term. The hypotheses are:

H_0_: *p_ij_* = *p_ji_* = cor (*u_it_*, *u_jt_*) = 0 for *i* ≠ *j*
H_1_: *p_ij_* = *p_ji_* ≠ 0 for some *i* ≠ *j*

where *p_ij_* is the pairwise correlation coefficient of the error term:

(4) $$p_{ij}=p_{ji}=\frac{\sum_{t=1}^{T}u_{it}u_{jt}}{{\left(\sum_{t=1}^{T}u_{it}^{2}\right)}^{1/2}{\left(\sum_{t=1}^{T}u_{jt}^{2}\right)}^{1/2}}$$

With the Pesaran test [33,34], Pesaran developed the following *CD* statistic, which is based on the LM statistic of Breusch and Pagan [36]:

(5) $$CD=\sqrt{\frac{2T}{N\left(N-1\right)}}\overset{N-1}{\underset{i=1}{\sum }}\overset{N}{\underset{j=i+1}{\sum }}{\hat{p}}_{ij}$$

This *CD* statistic is better than the LM statistic because the exact mean for the fixed numbers T and N includes heterogeneous/homogeneous and nonstationary models.

Friedman [37], who depended on the average Spearman’s rank correlation coefficient, developed the following statistic:

(6) $$R_{ave}=\frac{2}{N\left(N-1\right)}\overset{N-1}{\underset{i=1}{\sum }}\overset{N}{\underset{j=i+1}{\sum }}{\hat{r}}_{ij}$$

where *r_ij_* is the sample rank correlation coefficient, which is calculated from residuals, and *r_i,t_* is the rank matrix of *u_it_*:

(7) $$r_{ij}=r_{ji}=\frac{\sum_{t=1}^{T}\left\{r_{i,t}-\left(T+1/2\right)\right\}\left\{r_{j,t}-\left(T+1/2\right)\right\}}{\sum_{t=1}^{T}{\left\{r_{i,t}-\left(T+1/2\right)\right\}}^{2}}$$

Frees [38] developed the test statistic shown below, which can solve this issue by using the sum of squared rank correlation from the residual:

(8) $$R_{ave}^{2}=\frac{2}{N\left(N-1\right)}\overset{N-1}{\underset{i=1}{\sum }}\overset{N}{\underset{j=i+1}{\sum }}{\hat{r}}_{ij}^{2}$$

The test has a disadvantage in the case where T is small—it leads to poor Q distribution. When T is large, however, the test works well [39].

#### 3.2.2. Panel Unit Root Test

First and foremost, means of the panel unit root tests are employed to set the order of integration of the series. Next, if there is evidence suggesting non-stationarity among the variables, the existing cointegrating relationships among them should be examined to advocate the above specifications. After that, causality among the four variables is analyzed by carrying out Granger causality tests.

Like financial and macroeconomic time series, energy series are ambiguous in the general literature. Thus, it is vital to examine the integration of mission series before conducting a test. A series is an integrated series of order zero I(0) if it is stationary [40]. On the other hand, a series is an integrated series of order one I(1) if it is nonstationary in the level in spite of its stationarity in the first difference. To test the appearance of unit roots in time series, Dickey-Fuller (DF) or Augmented Dickey-Fuller (ADF) tests are traditionally used [40]. Likewise, there is proof of developing panel-based unit root tests relying on the time series tests in the literature, e.g., [41,42]. Tests developed by Choi [42] are appropriated to examine integration between the variables of interest in a cointegration analysis when the time dimension of the sample differs from country to country. Thus, this paper follows Choi’s approach for testing the panel unit root test.

Choi [42] suggested the use of a Fisher test, which combines the significance levels from individual unit root tests using Fisher’s [43] results; the model of Choi [42] is presented below:

(9) $$P_{m}=\frac{1}{2\sqrt{N}}\sum_{i=1}^{N}(-2\ln p_{i}-2)$$

The null and the alternative hypotheses of the Fisher-ADF test are:

H_0_: *p_i_* = 0 for all *i*
H_1_: Allows for some (but not all) of the individual variables to have unit roots.

#### 3.2.3. Panel Cointegration Test

To test the appearance of long-run relationships between variables, the cointegration test is employed widely in the literature of time series. Based on Engle and Granger [44], there is cointegration between two nonstationary variables if these integration variables with similar orders have a linear combination with a lower order. For example, a linear combination of oil consumption and CO_2_ emissions is I(0) and of both of them is I(1); as a result, there is cointegration between two variables and a long-run relationship form that it is stationary in the long run, rather than having an ever-growing amount of difference, is created.

Cointegration tests are similar to individual unit root tests in the literature of time series when the time dimensions are short, since they suffer from low power. Panel techniques might be better for discovering cointegration relationships because pooled-level regression corporates are cross-sectional along with time series information while determining the cointegrating coefficients.

Kao [45] and Pedroni [46] suggested that panel cointegration tests are the same as the framework of Engle and Granger [44], which contains an examination of the stationarity of the residuals from regression levels. In addition, Westerlund [47] proposed that an error-correction panel cointegration test is suitable for the appearance of cointegration in the country as well as panel level. The test of Kao relies on the model as follows:

y_it_ = α_i_ + βx_it_ + e_it_ (10)

y_it_ = y_i,t−1_ + u_it_ (11)

x_it_ = x_i,t−1_ + v_it_ (12)

where i = 1, …, N; t = 1, …, T; α_i_ is the intercepts; β is the slope across i; e_it_ is the error term; and both y_it_ and x_it_ contain a unit root. Kao’s test was established to examine the existence of cointegration between y_it_ and x_it_ [45]. Equation (10) is designed employing the Least Square Dummy Variable (LSDV) and the residuals tested rely on the ADF equation as follows:

(13) $${\hat{e}}_{it}=p\hat{e_{i,t-1}}+\overset{p}{\underset{j=1}{\sum }}\gamma_{j}\hat{e_{i,t-j}}+v_{itp}$$

where *p* presents the number of the lags shown to conduct the residuals in uncorrelated Equation (13). The ADF test statistic is donated as a usual *t*-statistic when *p* = 1 in Equation (13), distributed asymptotically according to the normal standard. To examine whether there is the existence of cointegration between *x_it_* and *y_it_* according to the ADF test statistic, the alternative and null hypotheses can be expressed as H_0_: *p* = 1 and H_1_: *p* < 1, respectively.

Pedroni [46] improved on a different residual-based cointegration test relying on the null of non-cointegrated for heterogeneous panels. In Equation (14), Pedroni’s test differs from Kao’s test with regard to assuming p to be heterogeneous across cross-sections. The test statistic relies on separately estimating cointegration test statistics for each cross-section and, after that, averaging them to look for a cointegration test for the whole panel; therefore, it carries out well if the size of a sample has an adequate time horizon for each cross-section.

Pedroni’s model [46]:

(14) $$e_{it}=p_{i}e_{it-1}+\overset{p_{i}}{\underset{j=1}{\sum }}\Psi_{ij}\Delta e_{it-1}+v_{it}$$

Westerlund’s model [47]:

(15) $$\Delta y_{it}=\delta_{i}^{\prime }d_{t}+\alpha_{i}y_{it-1}+\lambda_{i}^{\prime }X_{it-1}+\overset{p_{i}}{\underset{j=1}{\sum }}\alpha_{ij}\Delta y_{it-j}+\overset{p_{i}}{\underset{j=0}{\sum }}\gamma_{ij}\Delta X_{it-j}+e_{it}$$

#### 3.2.4. Long-Run Estimates

The next stage continues determining the long-run relationship after there are cointegrations among the variables. Plenty of existing independent variables available for determining a cointegration vector employ panel data such as OLS (Ordinary Least Square), DOLS (Dynamic Ordinary Least Square), and FMOLS (Fully Modified Ordinary Least Square). Maeso-Fernandez et al. [48] stated that FMOLS, which is a non-parametric approach, takes responsibility for measuring the correlation between the first alternatives of independent variables and the error term along with the appearance of a constant term to solve reforms for serial correlations. Mehrara [49] suggested that DOLS is a parametric approach in which the lagged first-difference terms are determined clearly. In DOLS, the errors term is increased with lags, leads, and contemporaneous values of the independent variables. Determining the long-run equation by employing the ordinary least squares (OLS) method results in biased independent variables when the explanatory variables are not strictly exogenous so that the OLS independent variables cannot be employed for valid inferences in general. Pedroni [50] suggested FMOLS estimation, while Kao and Chiang [51] and Mark and Sul [52] proposed DOLS, as different methods of cointegration estimation in panel data. This paper uses the Pedroni model [46]:

(16) $$Y_{it}=\alpha i+\beta_{i}EC_{it}+\mu_{it};\;i=1,2,\ldots N\;\mathrm{and}\;t=1,2,\ldots ,T$$

where *Y_it_* denotes the dependent variable and EC denotes the vector of residual and different stationarity.

(17) $$Y_{it}=\alpha_{i}+\beta_{i}EC_{it}+\overset{k_{i}}{\underset{k=-k_{i}}{\sum }}\gamma_{ik}\Delta EC_{it-k}+\mu_{it};i=1,2,\ldots ,T$$

The FMOLS and DOLS estimators are presented below:

(18) $$\beta_{fmols}^{*}=N^{-1}\overset{N}{\underset{1}{\sum }}{\left(\overset{T}{\underset{t=1}{\sum }}{\left(EC_{it}-{\bar{EC}}_{i}\right)}^{2}\right)}^{-1}(\overset{T}{\underset{t=1}{\sum }}\left(EC_{it}-{\bar{EC}}_{i}\right)y_{it}^{*}-T_{\hat{\gamma i}})$$

(19) $$\beta_{dols}^{*}=N^{-1}\overset{N}{\underset{i=1}{\sum }}{\left(\sum_{t=1}^{T}Z_{it}Z_{it}^{i}\right)}^{-1}\left(\overset{T}{\underset{t=1}{\sum }}Z_{it}Y_{it}^{*}\right)$$

where *Z_it_* is the 2(*K* + 1) × 1 vector of regressors

(20) $$Z_{it}=\left\{\left(X_{i,t}-{\bar{X}}_{i}\right),\Delta X_{i,t-k},\ldots ,\Delta X_{i,t+k}\right\};{\tilde{Y}}_{i,t}=Y_{i,t}-{\bar{Y}}_{i}$$

#### 3.2.5. Panel Causality Test

Based on the Granger theory, if two I(1) series become cointegrated, they might be defined as being created by a mechanism of error correction. Nevertheless, the appearance of a cointegration relationship might not signify direct causal linkages among variables. To analyze the direct causal linkages one should carry out a panel-based vector error correction model (VECM). This model might be determined relying on the two-step procedure of Engle-Granger. Firstly, the long-run relationship is calculated through Equation (17) to build an error correction term (ECT) for the following step. An ECT is determined by one-period lagged residuals and the clue implies whether there are variables (one or two) adjusting deviation from the long-run relationship. Moreover, the coefficients on the ECT characterize fast changes of each variable’s deviations from the long-run equilibrium and are then eliminated. Secondly, the VECM is determined by the LSDV (least square dummy variable) estimation proposed by Bruno [53]. In this step, the two-equation VECM can be written as the following models:

(21) $$\Delta D_{it}=\theta_{li}+\lambda_{1}\varepsilon_{i,t-1}+\overset{m}{\underset{k=1}{\sum }}\theta_{11k}\Delta D_{i,t-k}+\overset{m}{\underset{k=1}{\sum }}\theta_{12k}\Delta ID_{i,t-k}+u_{lit}\lambda_{1}<0$$

(22) $$\Delta D_{it}=\theta_{2i}+\lambda_{2}\varepsilon_{i,t-1}+\overset{m}{\underset{k=1}{\sum }}\theta_{21k}\Delta ID_{i,t-k}+\overset{m}{\underset{k=1}{\sum }}\theta_{22k}\Delta D_{i,t-k}+u_{2it}\lambda_{2}<0$$

Equations (21) and (22) have endogeneity because lags of dependent variables present as independent variables and all lags of dependent variables contain unobserved fixed effects. Even though within the transformation the fixed effect estimation is eliminated, the endogeneity still exists. The endogeneity is a popular problem for dynamic panel data models and it results in biased coefficient results of dependent variables’ lags. To determine unbiased coefficients, Anderson-Hsiao [54] proposed instrumental variable (IV) estimation and Arellano-Bond [55] developed a generalized method of moments (GMM) estimation. These estimation techniques are conducted according to transformations of the models in Equations (21) and (22) by first altering them to get rid of individual fixed effects and, after that, employing past values of dependent variables, like instruments for endogenous variables. One disadvantage of these estimations is that their features hold only when N is really large and T is small in such a way that they are employed to micro panel data in general.

Nickell [56] claims that if T → ∞, the LSDV estimator will be suitable and will be biased to a trivial degree. Nonetheless, Judson and Owen [57] state that if T is smaller than 30, the LSDV estimator will have a bias of up to 20 percent in comparison with the true value coefficient of interest. They also point out that a bias-corrected LSDV works well in comparison with a GMM estimator or instrumental variable in a balance panel. Nevertheless, at the same time, Judson and Owen [57] stated that this method was limited because no one has developed this technique to implement it for unbalanced panels in such a way that they recommended employing one-step GMM by Arellano Bond [55] or AH estimator by Anderson-Hsiao [54] as the best alternative way when T is about 20. Their results relied on Monte Carlo simulation. Bruno [53] presented a formula for an LSDV estimator that calculated approximate bias and proposed an LSDV estimator to estimate bias-correction for unbalanced panels data when the average T across cross-sections is greater than or equal to 20 and when N is small. Because the LSDV estimator is not suitable without T → ∞, the bias-corrected LSDV employs and starts a suitable estimator, such as IV or GMM, and holds greater than 90 percent of the real bias of the LSDV estimator.

As a note for future studies, a potential nonlinear causality relationship among the variables should be considered (see, for example, Bai et al. [58,59] and Chow et al. [60]). In addition, advanced diagnostic check (see, for example, Hui et al. [61]) should be considered.

## 4. Empirical Results

### 4.1. Test of Presence of Cross-Section Dependence

From the test of cross-section dependence, all the tests have general null hypotheses H_0_: Cross-sectional independence and H_1_: Cross-sectional dependence.

From test results in two specifications, which are shown in Table 3, Pesaran statistics have a p-value of more than 5% which states that the null hypothesis is accepted. The results of Friedman are in line with Pesaran, this suggests that there is no cross-sectional dependence. In contrast, Free provides a rejection of null hypothesis at 1% level. This is different because both Pesaran and Friedman use the sum of the pairwise correlation coefficient of a residual matrix (such as LM statistic). This would make these tests weaker because of the disturbances with large positive and negative correlations, but they cancel each other out in the average. However, Frees [38] developed a test statistic that can solve this issue by using the sum of the squared rank correlation from the residual. Moreover, when T → ∞, the test works well, but the test has the disadvantage for the case when the T dimension is small, in which case it leads to poor Q distribution [39].

All the tests have their advantages and disadvantages. In this paper, the conflicting results were shown; however, the results from this test show us the way to choose the method in testing causality. For the case where all coefficients are the same across the cross-section, the Granger causality test would be employed. For the case of cross-section dependence, however, the Dumitrescu-Hurlin [62] approach, which allows for the differences of all coefficients across countries, should be used for testing the causality relationship. With the two statistics from Perasan and Friedman, we can conclude that there is no cross-section dependence in our sample, but the result from the Frees test indicates that there may be two potential cases. As such, this study was primarily based on the Granger test; however, we still ran an additional causality test, which was the Dumitrescu-Hurlin [62] approach, and then compared the results to ensure that they are robust.

### 4.2. Panel Unit Root Tests

Before utilizing the cointegration test to check the long-run relationship between economic growth (GDP), FDI, oil consumption (Oil), and CO_2_ emissions, this study used the unit root test for testing whether all variables are stationary and integrated of the same order. The purpose of conducting the unit root test is to identify the order of integration of the variables and to ensure that none of the underlined variables is I(2). The Choi test [42] uses the null hypothesis that the variables have a unit root or the variable is nonstationary, while the alternative hypothesis (H_1_) is having no unit root or the variable is stationary.

Table 4 presents the results of the unit root test for all variables in our model. At the 1 per cent significance level, all the variables are not stationary and have unit root in their level form. However, they all become stationary in their first difference form, I(1) in all 25 countries. Since the data are stationary in first difference, the cointegration test is taken for testing the existence of the long-run relationship between variables. If the test does not prove or show the existence of cointegration or long-run nexus, the Panel Vector Auto Regression (P-VAR) model should be applied to investigate the short-run effect [63,64]. In case of co-integration between variables, the FMOLS and the DOLS method can be used to estimate the long-run equilibrium coefficient before conducting the causality tests.

### 4.3. Panel Cointegration Test Results

Nonstationary variables at the significance level increase the potential for cointegration relationships among variables, the cointegration test needs to be used in this study to avoid spurious causality results, such as the spurious presence of causality or spurious absence of causality, as well as identifying the order of integration of the variables.

In the cointegration test, this study considers three methods: Kao [45], Pedroni [46], and Westerlun [47]. All three methods have the general null hypothesis H_0_: There is no cointegration. All results, which are shown in Table 5 above, rejected the null hypothesis at 1 per cent significance level. This states that there are long-run relationships between variables with all tests showing high statistical significance. There may be one or more cointegration relationships among them. Thus, the P-VAR method should not be used in this paper. In the next step, we estimate the long-run effect of variables using the FMOLS and DOLS models. For the final step, we use panel causality in both approaches, as we mentioned above, the Granger and Dumitrescu-Hurlin approaches [31,44,62], to check the uni-direction and bi-direction relationships between variables in order to have good estimators and recommendations for policymakers.

### 4.4. Regression Results

In order to estimate the long-run equilibrium relationship and to avoid the bias of Ordinary Least Squares (OLS hereafter) estimators of the parameters in the cointegrated panel series, this study employs panel cointegration FMOLS, which treats the endogeneity problem and its estimators are adjusted for serial correlation [50]. Moreover, this study also uses panel DOLS, which includes contemporaneous values, leads, and lags of the first difference of the regressors to correct the endogeneity problem and the serial correlation. While both specifications are alternative methods for panel cointegration [50,52], DOLS underperforms more than FMOLS because of the greater use of assumptions and the reduction in the degrees of freedom by using leads and lags [65]. Therefore, this study prefers the FMOLS results to the DOLS. As such, this study uses DOLS estimators as a confirmation in terms of direction and magnitude of a coefficient obtained by FMOLS. Furthermore, this study also runs regressions with time and without time trends for both specifications, since panel unit root tests state the series have popular stochastics trends and could lead to a bias in estimated results.

The results presented in Table 6 indicate that all the coefficients of lnGDP, lnGDP2, and lnGDP3 in both the FMOLS and DOLS techniques are statistically significant at the 1 per cent level (with and without trend) and the magnitude coefficient is very close. Thus, there is a strong long-run nexus between per capita income and CO_2_ emissions, especially, a non-linear relationship.

The first turning point of per capita income ranges from $122 to $149. This is quite low and from data of selected developing countries, it falls around the 1980s. The second turning point ranges from $1245 to $2797 across models; with the preferred FMOLS model, the difference is narrowed down to $1442 (with trend) and $2797 (without trend). The signs of lnGDP, lnGDP2, and lnGDP3 are negative, positive, and negative, respectively. This implies that there is an inverted N-shape for the nexus between GDP and CO_2_ emissions.

Additionally, Table 6 presents the impact of oil consumption on carbon emissions across models (except FMOLS with trend) as highly significant. This long-run elasticity ranges from 0.50 to 0.84, with the preferred model, FMOLS, as 0.71; this implies that a one percentage increase/decrease in oil consumption on average will lead to an increase/decrease in per capita CO_2_ emissions of 0.71 per cent.

From Table 6, the coefficients of lnFDI and lnFDI2 also have high statistical significance. In addition, the coefficients of lnFDI and lnFDI2 fall within a narrow range, from 0.514 to 0.574 and −0.0165 to −0.0149, suggesting that the nexus of FDI inflow and per capita CO_2_ emissions exhibits an inverted U-shape and this follows the traditional EKC shape. Using Formula (2) we calculated that the turning points of FDI are 35,817,222 and 30,963,577 for FMOLS without and with trend, respectively. From this turning point, the effect of FDI on carbon emissions will change from positive to negative.

### 4.5. Panel Causality Test

Based on the results of all tests above, especially the cointegration test, we continued to analyze causality through the pairwise directions, which tests for the uni-directional and bi-directional causality between variables, by the Granger causality tests for panel data. Hurlin and Venet [66] suggested that Granger causality testing, when applied to panel data, is better than time series data. This statement is explained through the following three reasons: (i) It controls the consistency between objects in the table data; (ii) it increases the reliability of the regression estimation; and (iii) it minimizes deviations in the use of research models and errors in time series data. From previous empirical studies, this is also the common method for testing causality but from the conflict test results between different statistics, this paper employs an additional approach, which is developed by Dumitrescu-Hurlin [62], for checking the robustness of the test results. As we mentioned above, the Granger test assumes all coefficients are the same across countries [31,44], but the other approach allows for differences across cross-sections [62]. The two approaches have similar null hypotheses H_0_: Variable 1 does not cause variable 2.

**Figure 2:**
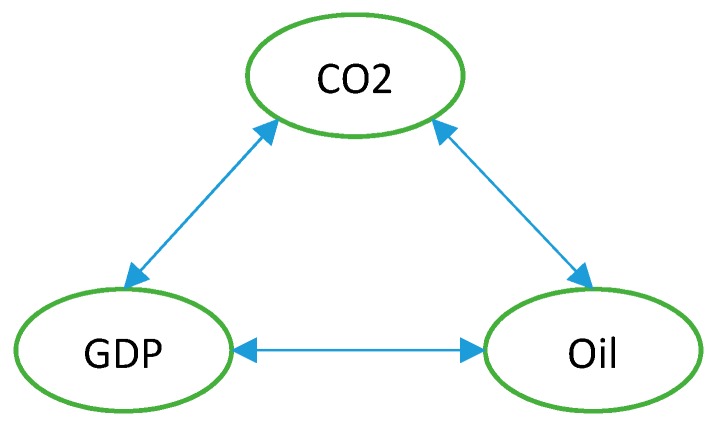
Summary of long-run Granger and homogeneous causality test.

Table 7 shows the full variables from our Equation (1), including all the variables that have bi-directional causality from both approaches. The full test results are presented in Appendix B and the summary for each test is presented in Appendix C and Appendix D. With the test result for our Equation (1), all variables strongly effect CO_2_ emissions, this supports the regression presented in Table 6. The results of both approach tests from Table 7 show that there are causal relationships between the main pairs of research models, as summarized Figure 2 below.

Statistical evidence from Granger showed that uni-directional causality occurs for pairs of variables: LnFDI and LnCO2; LnFDI2 and LnCO2. Another detail, the results of the Granger test also reject the null hypothesis that the above independent variables do not cause LnCO2 at the 1 and 5 per cent significance levels, meaning that the variables have a one-way effect on the LNCO2 variable. On the reverse side, there is not enough evidence about the existence of a causal relationship between LnCO2 and independent variables (LnFDI and LnFDI2) in the research model to reject the null hypothesis.

Moreover, the bi-directional causality relationship in the research sample appears in pairs of variables: LnGDP and LnCO2; LnGDP2 and LnCO2; LnGDP3 and LnCO2; LnOil and LnCO2, for both approaches. From both directions, all statistical evidence from four pairwise rejects the null hypothesis that there is no causal relationship between them at the significance level of 1% and 5%. Thus, the implication of research has shown that the three variables, CO_2_ emissions, income, and oil consumption, have a mutually causal relationship. This implies that an effort to reduce oil consumption will have greater effect on both environmental quality and economic growth. Thus, this causality showed an impossible trinity for policymakers.

### 4.6. Discussion

**Figure 3:**
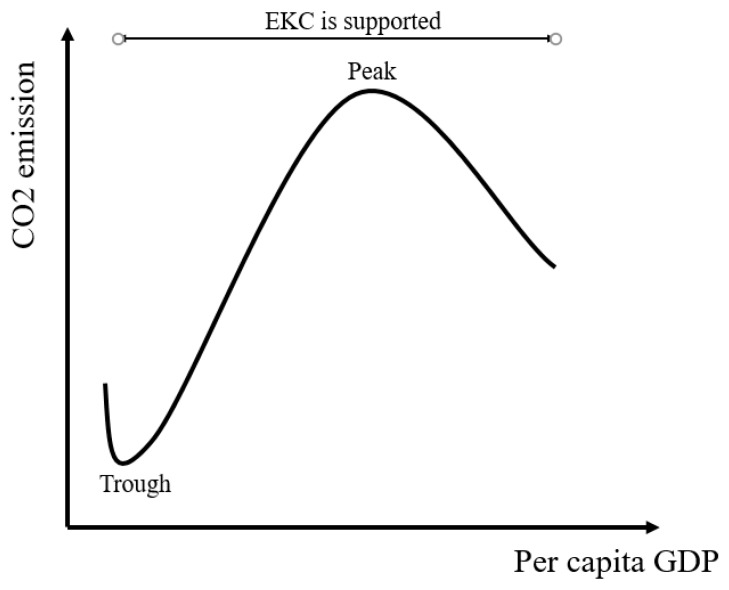
Schematic representation of income and CO_2_ emissions.

The results obtained from the FMOLS and DOLS reveal that all variables, which in the model include variables such as Oil, FDI, GDP, and their square and cubic versions, strongly effect carbon emissions. We found the relationship between per capita income and CO_2_ emissions does not follow the traditional EKC shape (inverted U), the results exhibit an inverted N-shape with the two turning points at $130 and $2797 (FMOLS estimators); this is quite new, from previous empirical studies. In order to explain the direction of this nexus, it should be divided into two stages, as presented in Figure 3.

For the first stage, the relationship between per capita income and CO_2_ emissions follows a normal U-shape. The plausible reason could be that all selected countries in Asia are currently developing countries and in the 1980s (the first stage) almost all these countries were poor and underdeveloped. The first turning point—the trough—coincides with the economy of almost all selected Asian countries at that time. When an economy was primarily based on agriculture (per capita income level is low), economic activities did not cause a bad and significant effect on the environment. However, after income increased to exceed the first threshold, when the countries started the industrialization process and adopted the open-door policies (e.g., Vietnam after 1986), the production grew rapidly, leading to an increase in energy consumption, especially oil consumption, and environmental degradation. Thus, the relationship followed a normal U-shape and this is consistent with the findings of Chandran and Tang [67], who used data from 1971 to 2008 from the ASEAN countries and found a normal U-shaped EKC for these countries.

With the particular emphasis of this study, that is, the relationship between per capita CO_2_ emissions and FDI inflow, the results presented in Table 6 show FDIs have a strong impact on CO_2_ emissions. This is not consistent with findings from previous empirical researches, such as Zhu et al. [18], Atici [27], Kirkulak et al. [24], Acharya [25], and Tang and Tan [20] who rejected the validity of the pollution heaven hypothesis. Contrary to the findings from these papers, this study finds that there is a non-linear long-run relationship between per capita CO_2_ emissions and FDI net inflow (1 per cent level in the preferred models with and without trend). The sign of these coefficients exhibits an inverted U-shape in both FMOLS specifications (with trend and without trend), thus the nexus between FDI and carbon emissions follows the shape of the EKC theory. These results support the findings from Zhou et al. [12]. Using the turning point, the peak of the FDI inflow is $35,817,222 (without trend) and $30,963,577 (with trend). These results appear to be robust and coincide with the data of selected countries in the 2010s. The initial effect of FDI inflow on CO_2_ emissions is positive, this implies that the Pollution heaven hypothesis is sufficiently supported. At the early stage of the foreign capital inflow, the host countries competed with other countries to attract more funds and investments by relaxing environmental standards. From that, the increase in capital inflow leads to the process of rapid industrialization, which provides a boost to the economy and an increase in production and energy consumption, which are the main causes of environmental problems.

However, the results also present evidence to confirm the view that, when the extreme point is achieved, the countries are moving into the second phase and that the nexus turns negative. This means, in the second phase, an increase in FDI could lead to a decrease in CO_2_ emissions. The possible reasons that could explain these findings are as follows. First, some countries in the region have achieved a certain level of pollution (such as air pollution from industries in Beijing, China in 2015 or Vietnam’s Formosa case in 2016). As such, environmental awareness requires that the countries must care about environmental quality and they need not trade-off between economic growth and environment by relaxing emission standards to attract more FDI. In contrast, the governments from these countries have raised discharge standards, requiring advanced technology for discharge treatment and limiting CO_2_ emissions for specific firms through auction permissions. As a result, the trend of improving environmental quality could reduce pollution in host countries and achieve sustainable growth goals. Second, the technology spillover benefits from developed countries to these developing countries raise efficiency in energy consumption and hence reduce CO_2_ emissions. This is in line with findings from Zhou et al. [12] and Lee [68]. Moreover, the inflow FDI leads to an increase in incentives from domestic R&D activities and a number of patents (empirical findings from Ito et al. [69] and Cheung and Ping [70]) and hence improves productivity, raises efficiency in input uses and energy consumption, and reduces emissions. Third, another way to reduce energy consumption is to use alternative sources. Clean and renewable energies, such as solar, wind, waves, electric, and geothermal energy, are currently in a great upward trend in developed countries that could dramatically reduce CO_2_ emissions.

In addition, oil consumption from the empirical results from this study reveal the strong impact on per capita CO_2_ emissions (FMOLS and DOLS without trend specifications) and the relationship is positive. This result is consistent with previous empirical findings that used total energy consumption in their studies, such as Chandran and Tang [67], Acaravci and Ozturk [71], and Ang [15]. The sign of the oil consumption coefficient is positive. This is in line with our expectation that the more oil use, the more CO_2_ emissions. The magnitude of this effect is quite large (near 1), suggesting that an effort to reduce oil consumption would have a greater effect in improving environmental quality.

Last but not least, the evidence from the causality test in both the Granger and Dumitrescu-Hurlin [62] approaches stated that there are bi-directional causalities between carbon emissions, income, and oil consumption. Thus, the policy that affects one of them will impact the two remaining ones. In order to have good and appropriate policies, they must simultaneously achieve reduction in environmental degradation and growth without trade-offs. The recommendation based on our findings is that policymakers should prioritize developing the policies that have reduced oil consumption without impacting on growth, such as technology improvement, they should use clean and renewable energies, and should boost the incentive for R&D activities to increase productivity through an increase in the efficiency of energy consumption and hence, achieve sustainable growth.

## 5. Conclusions

This study examines the effect of foreign direct investment on environment degradation in the Asian region and tests the validity of the traditional EKC curve. Findings from this study can be summarized as below.

First, in relation to the relationship between FDI and environmental degradation, we found that the pollution heaven hypothesis is valid in selected developing countries in the Asian region. FDI has a strong impact on the environment. This impact exhibits an inverted U-shape, which follows the traditional EKC curve. This means FDI can lead to an increase in emissions and also reduce the emissions. FDI leads to an increase in environment degradation at the first stage of economic growth and reduces it at the next stage. In order to have an appropriate and good policy for attracting FDI, the host country’s policymakers need to know clearly and exactly the optimal level of FDI for their country. The peak level of the inflow FDI, based on the threshold, can be estimated to ensure a good balance between environment and growth.

Second, with regard to the relationship between per capita income and environment degradation, we found the inverted N-shape in the selected developing countries in Asia, but the traditional EKC theory still has validity. As such, the trade-off between environment and growth in these countries from the past has existed to boost the performance of the economy.

Third, oil consumption is currently a major input of energy for industries and it has a strong effect on emissions in the selected developing countries in the Asia region. This implies that an effort to reduce oil consumption has dramatically provided a reduction in emissions or an improvement in environmental quality.

Fourth, the sustainable growth goal has the conflict or trade-off between growth and environment degradation, with bi-directional causalities between carbon emissions, income, and oil consumption. The change in one of these factors would lead to the change in both other factors and vice versa. Thus, policymakers should prioritize policies that reduce oil consumption and environment degradation but still boost economic growth. Our recommendation as to these kinds of policies is to encourage the incentives for R&D activities, such as technology, effectively improving energy use, and using alternative energies sources, which now are currently trending upwards in developed countries.

In conclusion, findings from this study support the view that policymakers in the Asia region should enhance the implementation of sustainable growth policies such as improving technology, capital, human resources, and the effective use of natural resources to ensure sustainable economic growth and development without negatively affecting the environment.

## Figures and Tables

**Table 1:** Variables description.

Variables	Expect Sign	Description	Definition	Sample Period	Sources
Carbon emissions (CO_2_)		Measured in Metric tons per capita	Carbon dioxide emissions are generated from burning fossil fuels and producing cement. They produce carbon dioxide emissions while using solid, liquid, and gas fuels.	1980–2016	The Integrated Carbon Observation System—ICOS [28]
Per capita income (GDP)	+/−	GDP per capita (current US$)	GDP per capita is defined as gross domestic product divided by population measured in the middle of the year.	1980–2016	WDI—World Bank [29]
Oil consumption (Oil)	+	Consumption of oil (kg of oil equivalent per capita)	Consumption of oil is transformed from daily barrels of oil equivalent to annual kg of oil equivalent by multiplying by 146.12 and 360 days, then dividing by population data from WDI.	1980–2016	U.S. Energy Information Administration [30]
FDI	+/−	Foreign direct investment, net inflows (BoP, current US$)	Foreign direct investment is mentioned as direct investment in economic reports. It includes equity, income reinvestment, and other types of capital.	1980–2016	WDI—World Bank [29]

**Table 2:** The descriptive statistics of all variables.

Variable *	CO_2_	FDI	GDP	GDP2	GDP3	FDI2	Oil
**Mean**	−0.2291	18.439	7.0918	51.5246	383.6231	351.1924	5.115
**Se(mean)**	0.0446	0.1184	0.0376	0.5603	6.4754	4.2362	0.0416
**p50**	−0.3403	18.6467	6.9983	48.976	342.7476	347.6984	5.2524
**Std. Dev.**	1.3565	3.3481	1.1102	16.5261	190.9969	119.8189	1.2584
**Variance**	1.8401	11.2097	1.2326	273.1113	36,479.82	14,356.56	1.5836
**Skewness**	0.2173	−0.4674	0.5558	1.0321	1.5435	0.2373	−0.385
**Kurtosis**	2.8909	4.2238	3.3666	4.4672	6.2551	2.8618	2.9596
**Sum**	−211.8964	14,751.2	6169.857	44,826.43	333,752.1	280,953.9	4669.983
**Range**	7.1295	24.0938	6.2255	95.361	1155.864	691.465	6.8506
**Min**	−3.5603	2.3026	4.5462	20.6677	93.9591	5.3019	0.9444
**Max**	3.5692	26.3963	10.7717	116.0288	1249.823	696.7669	7.795
**ID (n)**	25	25	25	25	25	25	25
**T-bar**	37	32	34.8	34.8	34.8	32	36.52
**Observations (*N*)**	925	800	870	870	870	800	913

Note: * All the variables are expressed in natural logarithm.

**Table 3:** Sectional independence tests.

Tests	Pesaran		Friedman		Frees	
	CD Test	*p*-Value	CD	*p*-Value	CD (Q)	*p*-Value
RE model	1.89	0.06	32.12	0.12	6.80 ***	0.00
FE model	1.92	0.06	29.83	0.19	6.92 ***	0.00

Note: FE and RE stand for fixed effects and random effects models, respectively. *** stated statistical significance at 1% level.

**Table 4:** Root test results.

Variable	Level		First Difference		Conclusion
	Lag Length	*p*-Value	Lag Length	*p*-Value	
CO_2_	1	0.99	1	0.00	I(1)
GDP	1	1.00	1	0.00	I(1)
FDI	1	0.64	1	0.00	I(1)
Oil	1	0.94	1	0.00	I(1)
GDP^2^	1	1.00	1	0.00	I(1)
GDP^3^	1	1.00	1	0.00	I(1)
FDI^2^	1	0.92	1	0.00	I(1)

**Table 5:** Cointegration tests.

Panel A: Kao [45]	CO_2_	*p*-Value
**Modified Dickey-Fuller t**	−3.33 ***	0.00
Dickey-Fuller t	−3.64 ***	0.00
Augmented Dickey-Fuller t	−2.69 ***	0.00
Unadjusted modified Dickey-Fuller t	−4.81 ***	0.00
Unadjusted Dickey-Fuller t	−4.27 ***	0.00
**Panel B: Pedroni [46]**		
Modified Phillips-Perron t	1.96 **	0.03
Phillips-Perron t	−3.49 ***	0.00
Augmented Dickey-Fuller t	−3.91 ***	0.00
**Panel C: Westerlun [47]**		
Variance ratio	−1.83 **	0.03

Note: ***, ** the rejection of null hypothesis of no cointegration is statistically significant at 1% and 5% levels, respectively

**Table 6:** Results.

Dependent Variable: lnCO_2_	FMOLS		DOLS	
	Without Trend	With Trend	Without Trend	With Trend
lnGDP	−8.836 ***	−10.41 ***	−9.811 ***	−10.20 ***
	(1.876)	(1.796)	(0.914)	(0.899)
lnGDP2	1.465 ***	1.799 ***	1.632 ***	1.744 ***
	(0.311)	(0.307)	(0.149)	(0.149)
lnGDP3	−0.0763 ***	−0.0993 ***	−0.0868 ***	−0.0962 ***
	(0.017)	(0.018)	(0.008)	(0.008)
lnFDI	0.574 ***	0.514 ***	−0.103	−0.0576
	(0.138)	(0.129)	(0.105)	(0.103)
lnFDI2	−0.0165 ***	−0.0149 ***	0.00136	0.000333
	(0.004)	(0.003)	(0.003)	(0.003)
lnOil	0.712 ***	0.181	0.840 ***	0.505 ***
	(0.046)	(0.229)	(0.031)	(0.100)
Time trend		0.0530 **		0.0341 ***
		(0.023)		(0.010)
Constant	8.338 **	11.98 ***	16.19 ***	16.41
	(3.684)	(3.599)	(1.919)	(0.000)
**Turning points—TNP ($US)**				
1st TNP of GDP_PC_	130	122	149	142
2nd TNP of GDP_PC_	2797	1442	1869	1245
Peak of FDI_IF_	35,817,222	30,963,577		
R-squared	0.977	0.984	0.996	0.996

Standard errors are in parentheses; FDI_IF_ denotes net inflow; GDP_PC_ denote per capita income. *** *p* < 0.01, ** *p* < 0.05.

**Table 7:** Causality test.

Null Hypothesis	Granger Causality		Dumitrescu-Hurlin		
	F-Statistic	Prob.	W-Stat.	Zbar-Stat.	Prob.
LNFDI does not cause LNCO2	6.88	0.00	5.34	6.58	0.00
LNCO2 does not cause LNFDI	1.41	0.24	6.39	8.78	0.00
LNFDI2 does not cause LNCO2	7.70	0.00	5.62	7.18	0.00
LNCO2 does not cause LNFDI2	0.50	0.61	6.32	8.62	0.00
LNGDP does not cause LNCO2	5.56	0.00	4.43	4.76	0.00
LNCO2 does not cause LNGDP	3.33	0.04	3.88	3.62	0.00
LNGDP2 does not cause LNCO2	6.36	0.00	4.23	4.35	0.00
LNCO2 does not cause LNGDP2	3.37	0.04	3.71	3.25	0.00
LNGDP3 does not cause LNCO2	6.52	0.00	4.07	4.02	0.00
LNCO2 does not cause LNGDP3	3.81	0.02	3.59	3.00	0.00
LNOILCON does not cause LNCO2	3.34	0.04	4.05	4.11	0.00
LNCO2 does not cause LNOILCON	14.86	0.00	6.67	9.79	0.00
LNOILCON does not cause LNGDP	6.59	0.00	3.94	3.73	0.00
LNGDP does not cause LNOILCON	10.68	0.00	10.98	18.55	0.00
LNOILCON does not cause LNGDP2	5.78	0.00	3.81	3.44	0.00
LNGDP2 does not cause LNOILCON	10.35	0.00	9.96	16.41	0.00
LNOILCON does not cause LNGDP3	5.85	0.00	3.68	3.19	0.00
LNGDP3 does not cause LNOILCON	9.38	0.00	8.95	14.28	0.00

**Figure A1:**
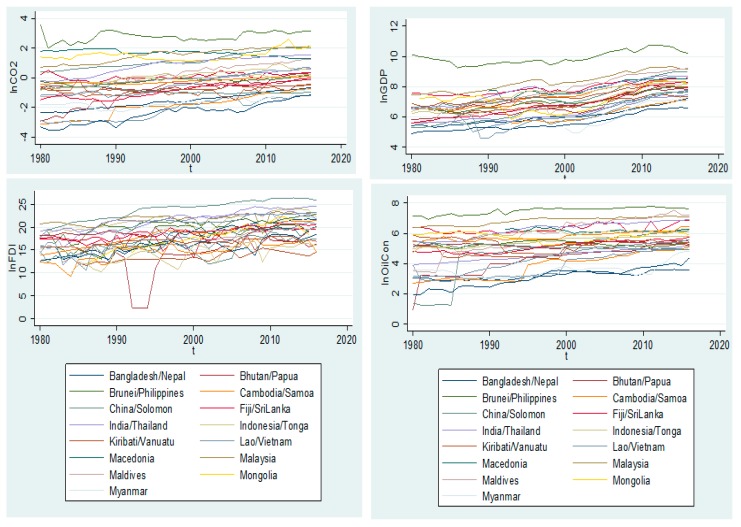
Line chart for the historical trend of variables: lnCO2, lnGDP, lnFDI, and lnOilCon over 1980–2016.

**Figure A2:**
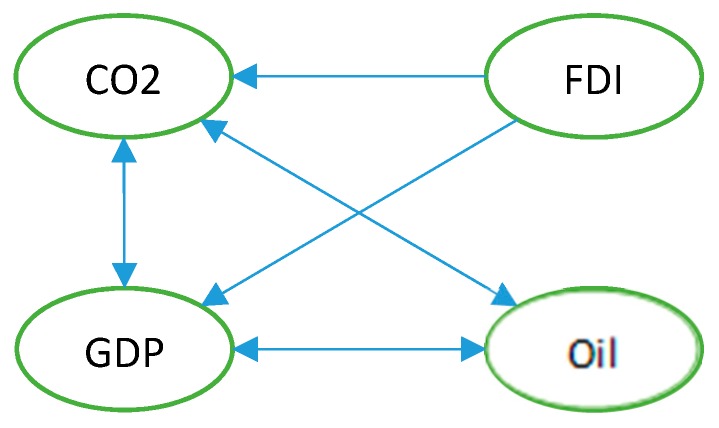
Summary of long-run Granger causality test.

**Figure A3:**
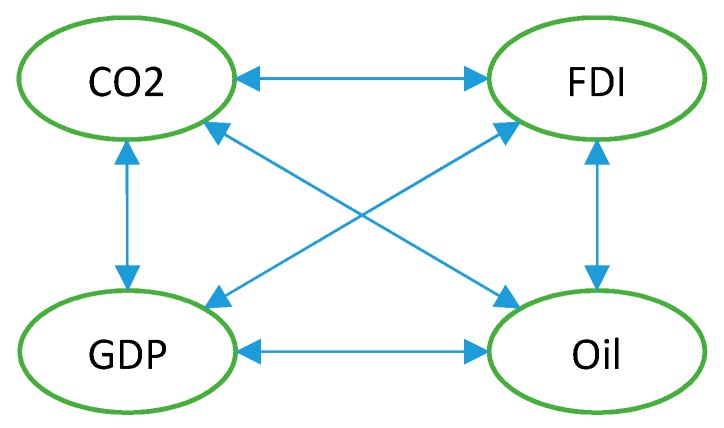
Summary of long-run homogeneously causality test.

## Appendix B. Full Results from Causality Test

**Table A1:** Causality test results from both approaches for all variables.

Null Hypothesis	Granger Causality		Dumitrescu-Hurlin		
	F-Statistic	Prob.	W-Stat.	Zbar-Stat.	Prob.
LNFDI does not cause LNCO2	6.88	0.00	5.34	6.58	0.00
LNCO2 does not cause LNFDI	1.41	0.24	6.39	8.78	0.00
LNFDI2 does not cause LNCO2	7.70	0.00	5.62	7.18	0.00
LNCO2 does not cause LNFDI2	0.50	0.61	6.32	8.62	0.00
LNGDP does not cause LNCO2	5.56	0.00	4.43	4.76	0.00
LNCO2 does not cause LNGDP	3.33	0.04	3.88	3.62	0.00
LNGDP2 does not cause LNCO2	6.36	0.00	4.23	4.35	0.00
LNCO2 does not cause LNGDP2	3.37	0.04	3.71	3.25	0.00
LNGDP3 does not cause LNCO2	6.52	0.00	4.07	4.02	0.00
LNCO2 does not cause LNGDP3	3.81	0.02	3.59	3.00	0.00
LNOILCON does not cause LNCO2	3.34	0.04	4.05	4.11	0.00
LNCO2 does not cause LNOILCON	14.86	0.00	6.67	9.79	0.00
LNOILCON does not cause LNGDP	6.59	0.00	3.94	3.73	0.00
LNGDP does not cause LNOILCON	10.68	0.00	10.98	18.55	0.00
LNOILCON does not cause LNGDP2	5.78	0.00	3.81	3.44	0.00
LNGDP2 does not cause LNOILCON	10.35	0.00	9.96	16.41	0.00
LNOILCON does not cause LNGDP3	5.85	0.00	3.68	3.19	0.00
LNGDP3 does not cause LNOILCON	9.38	0.00	8.95	14.28	0.00
LNFDI2 does not cause LNFDI	20.19	0.00	3.52	2.79	0.01
LNFDI does not cause LNFDI2	7.48	0.00	3.38	2.50	0.01
LNGDP does not cause LNFDI	0.36	0.70	9.34	14.62	0.00
LNFDI does not cause LNGDP	3.09	0.05	6.48	8.75	0.00
LNGDP2 does not cause LNFDI	0.32	0.73	8.98	13.89	0.00
LNFDI does not cause LNGDP2	2.95	0.05	5.83	7.43	0.00
LNGDP3 does not cause LNFDI	0.30	0.74	8.75	13.41	0.00
LNFDI does not cause LNGDP3	2.74	0.07	5.14	6.01	0.00
LNOILCON does not cause LNFDI	0.73	0.48	5.75	7.44	0.00
LNFDI does not cause LNOILCON	1.12	0.33	3.43	2.61	0.01
LNGDP does not cause LNFDI2	1.42	0.24	8.74	13.39	0.00
LNFDI2 does not cause LNGDP	3.99	0.02	5.92	7.61	0.00
LNGDP2 does not cause LNFDI2	1.32	0.27	8.27	12.43	0.00
LNFDI2 does not cause LNGDP2	3.81	0.02	5.54	6.83	0.00
LNGDP3 does not cause LNFDI2	1.26	0.29	8.01	11.90	0.00
LNFDI2 does not cause LNGDP3	3.54	0.03	5.08	5.89	0.00
LNOILCON does not cause LNFDI2	0.86	0.42	5.57	7.06	0.00
LNFDI2 does not cause LNOILCON	1.58	0.21	3.24	2.21	0.03
LNGDP2 does not cause LNGDP	0.02	0.98	2.58	0.86	0.39
LNGDP does not cause LNGDP2	0.66	0.51	2.47	0.63	0.53
LNGDP3 does not cause LNGDP	0.10	0.91	2.54	0.78	0.44
LNGDP does not cause LNGDP3	2.52	0.08	2.36	0.41	0.69
LNGDP3 does not cause LNGDP2	1.25	0.29	2.45	0.60	0.55
LNGDP2 does not cause LNGDP3	2.98	0.05	2.39	0.46	0.64
